# Zero-shot denoising of microscopy images recorded at high-resolution limits

**DOI:** 10.1371/journal.pcbi.1012192

**Published:** 2024-06-10

**Authors:** Sebastian Salwig, Jakob Drefs, Jörg Lücke

**Affiliations:** Machine Learning Lab, Department of Medical Physics and Acoustics, School of Medicine and Health Sciences, University of Oldenburg, Oldenburg, Germany; University of Virginia, UNITED STATES

## Abstract

Conventional and electron microscopy visualize structures in the micrometer to nanometer range, and such visualizations contribute decisively to our understanding of biological processes. Due to different factors in recording processes, microscopy images are subject to noise. Especially at their respective resolution limits, a high degree of noise can negatively effect both image interpretation by experts and further automated processing. However, the deteriorating effects of strong noise can be alleviated to a large extend by image enhancement algorithms. Because of the inherent high noise, a requirement for such algorithms is their applicability directly to noisy images or, in the extreme case, to just a single noisy image without *a priori* noise level information (referred to as blind zero-shot setting). This work investigates blind zero-shot algorithms for microscopy image denoising. The strategies for denoising applied by the investigated approaches include: filtering methods, recent feed-forward neural networks which were amended to be trainable on noisy images, and recent probabilistic generative models. As datasets we consider transmission electron microscopy images including images of SARS-CoV-2 viruses and fluorescence microscopy images. A natural goal of denoising algorithms is to simultaneously reduce noise while preserving the original image features, e.g., the sharpness of structures. However, in practice, a tradeoff between both aspects often has to be found. Our performance evaluations, therefore, focus not only on noise removal but set noise removal in relation to a metric which is instructive about sharpness. For all considered approaches, we numerically investigate their performance, report their denoising/sharpness tradeoff on different images, and discuss future developments. We observe that, depending on the data, the different algorithms can provide significant advantages or disadvantages in terms of their noise removal vs. sharpness preservation capabilities, which may be very relevant for different virological applications, e.g., virological analysis or image segmentation.

## Author summary

For any image (e.g., recorded using digital photography or electron microscopy), there is a limiting resolution for which the image still looks sharp. Beyond this resolution (by further zooming in), an image becomes increasingly noisy. Resolution limits may be improved technically (bigger lenses, more light, longer exposure times etc.). Alternatively, or additionally, modern denoising algorithms can be applied after the image is recorded. Here we investigate state-of-the-art such algorithms with many of them being developed in field of Machine Learning. The algorithms work by first learning representations of images (e.g., edges, textures etc.), which they then use to remove the noise from an image. To be applicable at high resolutions, algorithms need to be able to learn from noisy images. If just one noisy image is available for learning without *a priori* noise level information, the task is also referred to as blind zero-shot denoising. Importantly, we argue that the implicit goal of denoising is noise removal without majorly diminishing image sharpness. All the here investigated blind zero-shot algorithms are consequently evaluated based on both: noise removal and sharpness preservation. We observed salient strengths and weaknesses of different approaches for different image types, which can significantly impact different tasks.

This is a *PLOS Computational Biology* Benchmarking paper.

## Introduction

Electron and conventional microscopy can visualize fine details from the micrometer to subnanometer range and are used in biology, medical science, and many other fields. High resolution approaches such as transmission electron microscopy (TEM) have, for instance, significantly contributed to our understanding of how a given virus infects a cell, how it binds to cells and different tissues, and how it spreads in the body [1, 2]. For the analysis of virus infections, it is of high importance to appropriately visualize fine details at scales of viruses and cells. Otherwise, recorded structures can be confused with viruses, leading to undesirable misinterpretations of infection scenes, as was discussed, e.g., in the context of SARS-CoV-2 infections [3].

The quality of TEM images are influenced by many factors. For instance, only a finite electron dose may be used to avoid damaging the sample. Other factors include stability of the microscope and sample stage as well as electrostatic and magnetic field noise, which may lead to the exclusive use of short exposure times in order to not compromise the resolution [4]. Therefore, TEM images near their highest resolution contain significant amounts of noise. As reported in [5], TEM images are affected by three different types of noise: (i) structural noise resulting from the carbon in the background, (ii) Poisson noise due to fluctuations in the number of emitted electrons, and (iii) digitization noise caused by the CCD camera or other scanning devices. Challenges regarding image interpretation and processing due to inherent noise also arise with other microscopy modalities including fluorescence microscopy (FM). In FM images, main noise sources include fluctuations of the photons and digitization noise, which are typically modeled using Poisson and Gaussian distributions, respectively (compare, e.g., [6, 7]).

**Fig 1:**
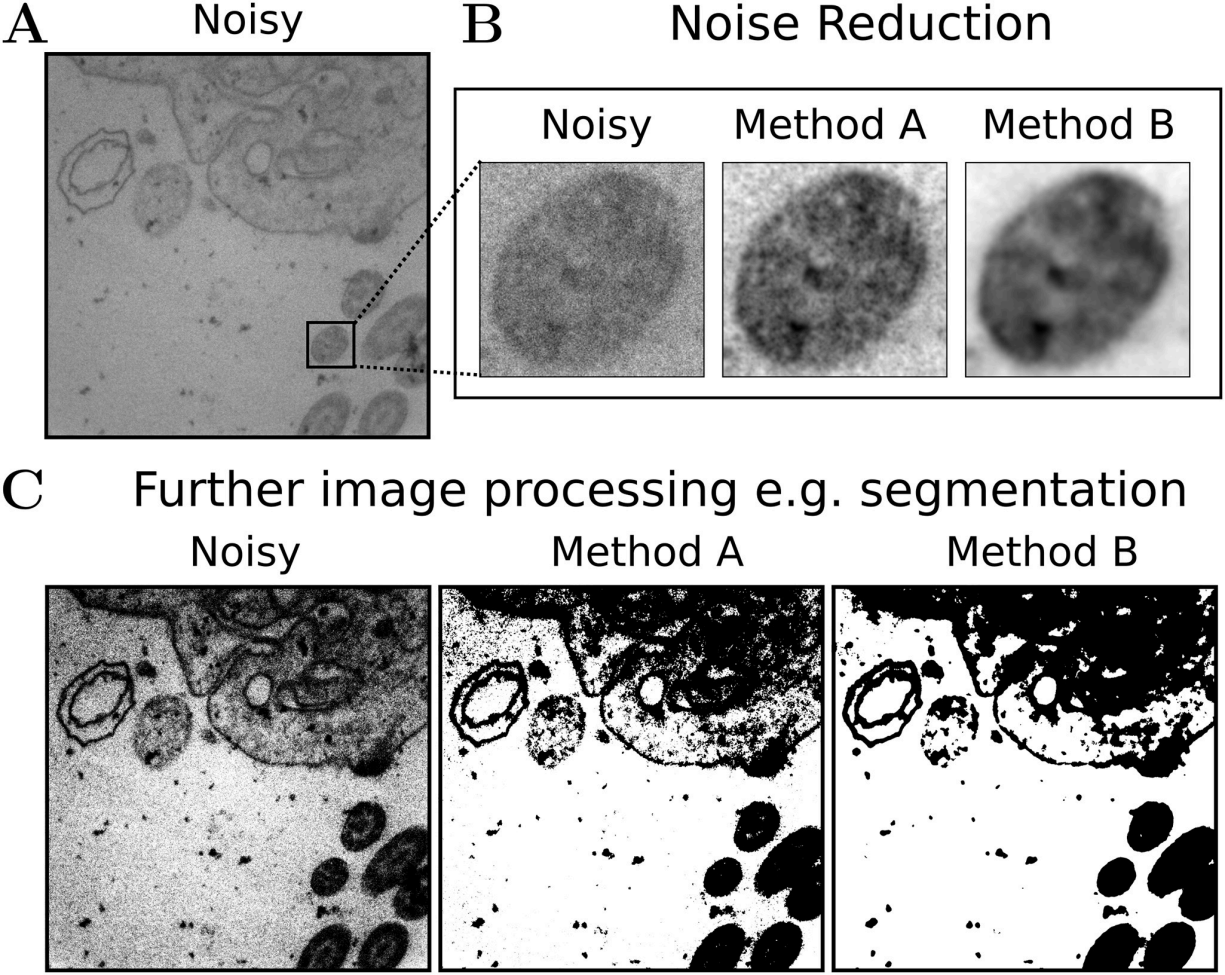
Comparison of noise reduction and blur. A: Noisy TEM image of cilia. B: Comparison of an image section of the noisy image, and two denoised versions. While method A reconstructs a sharper image compared to method B (blur effect_*A*_ = 0.34, blur effect_*B*_ = 0.68, lower is better), method B reduces noise more strongly (PSNR_*A*_ = 28.29, PSNR_*B*_ = 32.44; higher is better). C: Segmentation masks generated with Otsu’s method [11] based on the noisy image and the reconstructions of method A and B.

To address the problem of high noise, algorithms for noise removal are a very established tool (compare, e.g. [8]). These algorithms aim at reducing the noise while preserving the sharpness and structures such as edges, corners or other features of the original image. However, there is often a tradeoff between the amount of noise removed on the one hand and the loss of details/sharpness on the other [9, 10]. The definition of a ‘good’ denoised TEM or FM image is very subjective and depends on the application. For instance, strong denoising may achieve a ‘good’ quantitative assessment in terms of signal-to-noise improvement, but may be unsuitable for analysis from a virologist’s perspective because the processed image may be perceived as overly smooth, important details may be lost, or new artifacts may be generated. On the other hand, a strongly denoised and potentially blurred image may be suitable for further image processing tasks such as segmentation, as long as structures of objects important for the task are still preserved (compare Fig 1).

Features based on which the large body of existing denoising algorithms can be distinguished include, among others, the strategy applied for noise removal and the amount of *a priori* information an algorithm requires to denoise a given image. Block-Matching and 3D filtering (BM3D [12]), or Weighted Nuclear Norm Minimization (WNNM [13]), for instance, represent two prominent examples of approaches in the category of non-local image processing methods, that exploit image patch similarities within the image that is supposed to be restored. Both of these algorithms are tailored to Gaussian noise, and they assume the Gaussian noise level to be known *a priori*. BM3D has been extended in various ways, for instance to be applicable in the case of Poisson noise [14, 15]. Another filtering approach applicable to microscopy images include the Wavelet Based Background and Noise Subtraction (WBNS) algorithm [16]. Similar to BM3D or WNMM, this algorithm requires some form of *a priori* knowledge in order to be applicable (i.e., WBNS requires as input parameter the full width at half maximum of the microscope’s point spread function). Data-driven approaches form another category of algorithms, in which denoising models are learned from suitable data, for example large image databases. Widely used are discriminative models that are trained to learn a direct mapping from noisy to non-noisy images using a parameterized function in the form of a deep neural network (DNN). Supervised such algorithms that are trained based on large sets of paired examples of noisy and respective non-noisy images currently represent the state-of-the-art in a range of standard image restoration benchmarks (e.g., [17–20]). Besides attenuation of Gaussian noise, supervised denoising models were found to be highly effective also for a range of more complex types of noise (e.g., [21, 22]). However, with realistic microscopic image data, as considered in this study, the application of standard supervised algorithms poses a challenge or is not possible due to the lack of sufficiently noise-free training images. Similar applies to unsupervised methods that (at least in practice) leverage clean data for training (e.g., [23, 24]). Waiving the requirement of clean training data for supervised DNNs has been subject to current research: Evaluated on benchmarks with standard test images, Noise2Noise [25] (N2N), for instance, has been shown to improve the performance of BM3D-based denoisers while being trained on noisy data (and without requiring the noise level *a priori*). A prerequisite of N2N is, however, that a training dataset with different noisy realizations of the same underlying clean image can be constructed. Such an in practice usually unrealistic assumption motivated follow-up work in the form of Noise2Void [26] (N2V) which dropped the requirement of paired noisy images as in N2N training. On standard macroscopic test images, N2V showed competitive denoising performance, yet peak-signal-to-noise ratios (PSNRs) reported were not on par with BM3D and N2N [26]. Examples such as N2N or N2V highlight that direct applicability to noisy images has emerged as an increasingly important feature for data-driven denoising algorithms, and ideas exploited by these methods have been taken up in various ways [27–30], e.g., using a specific form of downsampling to derive a training data set from a single noisy input etc. (entitled ‘chequerboard downsampling’; Noise2Fast [31]).

A further, very established class of approaches is based on probabilistic generative models which have shown to provide powerful tools for a variety of image enhancement tasks including denoising, deblurring or inpainting [23, 32–35]. Generative approaches have also successfully been used, e.g., to decrease EM scan time and electron beam exposure [36] using training settings for inpainting or to model a SARS-CoV-2 binder based on 3D coordinates [37]. A foundational element is a mathematical description in terms of a probability density model of the data generating process which incorporates explicit noise assumptions. Furthermore, and unlike discriminate approaches, generative methodologies first learn a probabilistic representation of the data, which can subsequently be used for estimating the underlying non-noisy image (details under Materials and methods). Generative approaches are, usually, well suited for task settings with few data or few or no label information, i.e., they can naturally be trained on noisy data in an unsupervised way [32, 35, 38–40]. Hence, such a property makes them appealing tools in the context of microscopy image denoising.

With this paper, we aim at evaluating latest developments in the field of unsupervised image denoising using recent microscopy imaging datasets including, most prominently, very recent TEM images of SARS-CoV-2 infection scenes [41], TEM images of cilia [42], as well as fluorescence microscopy images of a recent benchmark on unsupervised denoising with deep generative models [30, 43] (details under Datasets). The concrete algorithms we here consider are (see Algorithms for details and Table 1 for an overview): median filtering (compare, e.g., [8]), BM3D [12] in conjunction with an auxiliary blind noise level estimator [44], BM3D with variance stabilizing transformation (VST+BM3D [14]), iterative BM3D with variance stabilizing transformation (I+VST+BM3D [15]), N2V, Noise2Fast (N2F [31]), Self2Self (S2S [29]), DivNoising (DivN [30]), Evolutionary Spike-and-Slab Sparse Coding (ES3C [45]), and a Poisson Mixture Model (PMM; compare e.g. [46]). Note that while BM3D itself is a non-blind zero-shot algorithm (as it requires *a priori* noise level information), all of the here considered variants are blind zero-shot approaches.

Our evaluations focus on the setting in which algorithms are provided with only the single noisy microscopy image that has to be denoised, excluding any *a priori* knowledge about the noise (also referred to as a blind zero-shot setting; compare [47–49]). Such benchmark conditions have also been considered in the context of other image restoration tasks such as super resolution [47, 50, 51]. Data representations learned based on single observations (such as a single noisy image) often occur naturally whenever data acquisition is difficult or the objects in the image are very rare. As pointed out in work by Laine *et al*. [52], a data-driven approach is likely to generate artifacts in the reconstructed image if the approach was trained on images with content statistics significantly different from the statistics of the image to be restored. Related to this point, it has (conversely) been argued that learning from single images can be advantageous as intrinsic image statistics contain higher quality information compared to statistics learned from large image corpora [47].

In a variety of denoising benchmarks, performance is commonly assessed based on PSNR values, which are indicative about the mean squared error (MSE) between the processed and target image in relation to the maximal image pixel value. The PSNR is a well established measure and is easy to compute. However, in several cases this evaluation metric performs poorly in reflecting human perception. Wang *et al*. [53, 54], for instance, discussed examples in which images with different distortions that obviously present different visual quality, achieved approximately the same MSE (and thus PSNR). Ndajah *et al*. [55] showed another example where the MSE was not able to properly capture the distortions due to blurring. Motivated by such contributions, this paper presents denoising performance evaluations that do not focus exclusively on noise suppression in terms of MSE/PSNR, but simultaneously inspect measures indicative about both noise suppression as well as image sharpness. A particular challenge for the here considered datasets with real microscopy images is thereby posed by the absence of ground-truth information (i.e., no ‘clean’ image data), implying that not only the algorithms themselves but also the evaluation measures need to be applicable on the basis of a single noisy image.

## Results

### Denoising TEM images of SARS-CoV-2 infected cell cultures

**Fig 2:**
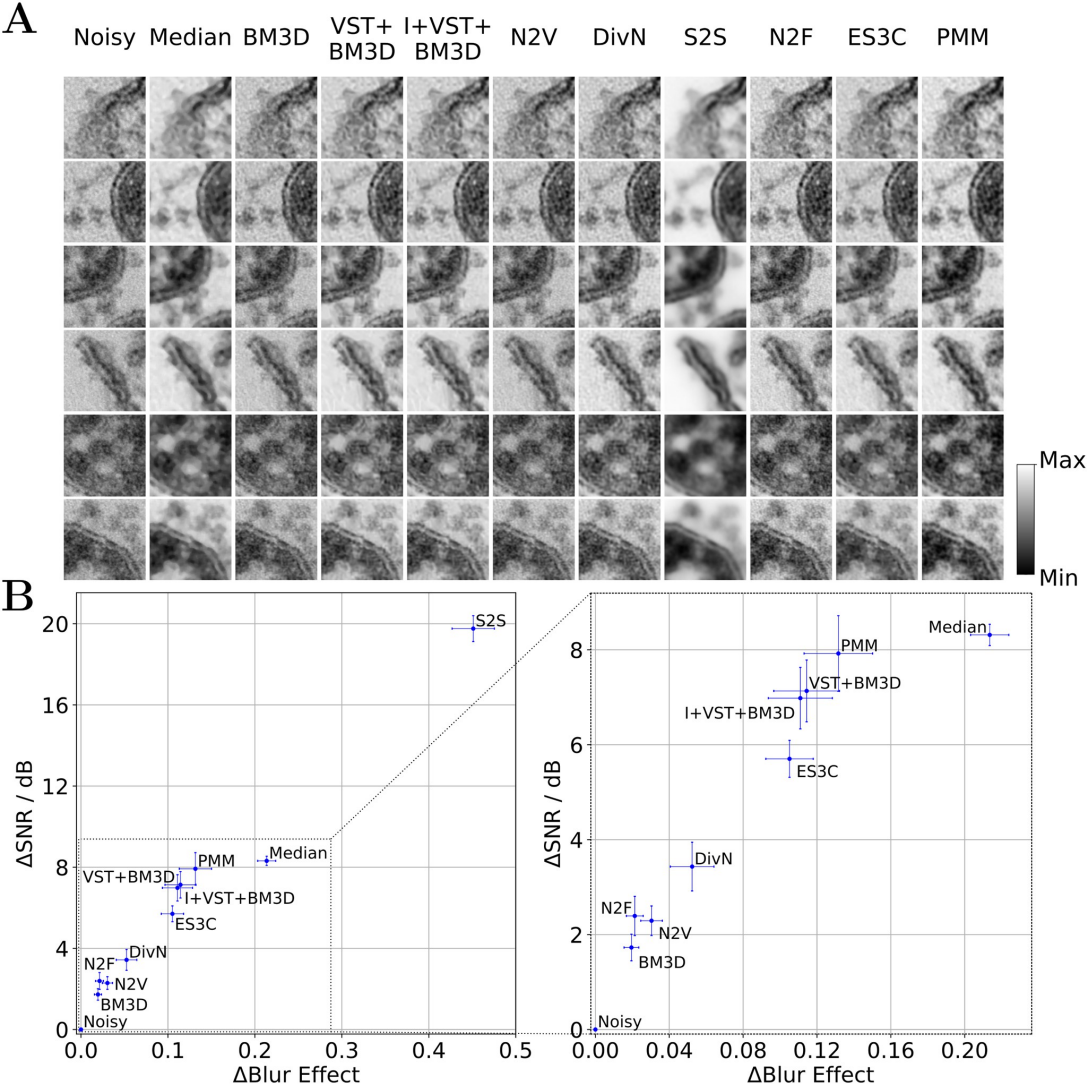
Results for blind zero-shot denoising TEM images of SARS-CoV-2 infected cell cultures. A: Qualitative comparison of denoised image sections showing virus and cell structures obtained with the investigated algorithms (image sections are scaled locally to fill the color range). B: Quantification of the denoising performance in terms of SNR in dB based on hand-labeled signal and background regions and blur effect (SNR and blur effect plotted against each other). The left subfigure displays the performance of all algorithms, while the right subfigure provides an enlarged view of the region populated by most algorithms. For both the SNR and blur effect measure, we measured the difference (delta) between the values obtained for the reconstructed and the noisy image and here depict averages together with standard errors of the mean (SEM) over the nine considered test images.

We first investigated blind zero-shot denoising of nine images selected from a publicly available dataset [41] containing TEM recordings of SARS-CoV-2 viruses in Vero cell cultures (see Datasets for details). In order to apply BM3D, VST+BM3D, I+VST+BM3D, N2V, S2S, N2F and DivN to these images, we used the implementations that were made publicly available alongside the official publications, and used the default hyperparameters of those implementations (details in S2 Text). All investigated algorithms were applied individually to each test image. Due to the lack of non-noisy reference images, PSNR values could not be computed. Therefore, we adopted a no-reference metric for noise removal and performed a signal-to-noise quantification based on hand-labeled signal and background regions [28]. We contrasted this noise metric with a no-reference blur metric [10]. The results of the experiment are depicted in Fig 2.

In Fig 2B, a tradeoff between SNR and blur effect achieved by the investigated algorithms can be observed. A significant difference exists between the values of S2S and the other investigated methods. While S2S results in the by far highest SNR (indicating strongest noise suppression), the algorithm also yields the highest blur effect score (indicating most severe blurring of the image). Median filtering achieves the second highest SNR, which is accompanied by the second highest value for the blur effect. After S2S and median filtering, the Poisson-based methods, including PMM, VST+BM3D, and I+VST+BM3D, remove the most noise according to SNR. The SNR and blur effect values of the Poisson-based methods are very close to each other. While ES3C achieves comparable results in terms of the blur effect measure, the algorithm achieves a slightly lower SNR than the Poisson-based methods. The remaining methods, that like ES3C assume additive noise (i.e. DivN, N2F, N2V and BM3D), offer the lowest noise suppression in terms of SNR; at the same time, they achieve the lowest blur effect values.

### Denoising of short-exposure TEM images of cilia

**Fig 3:**
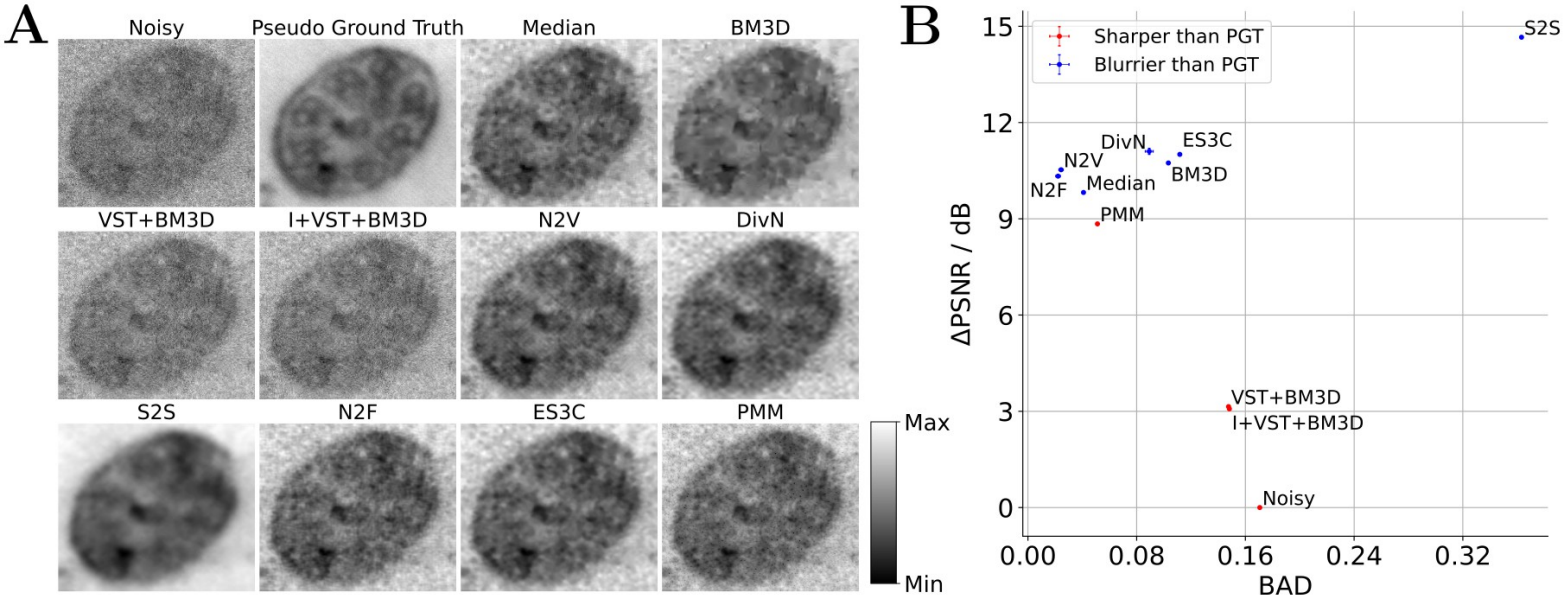
Results for blind zero-shot denoising short exposure TEM images of cilia (benchmark adopted from Bajic *et al*. [42]). A: Qualitative comparison of denoised image sections showing a cilium (image sections chosen to match Fig 3 in [42]; image sections are scaled locally to fill the color range). B: Quantification of the denoising performance in terms of PSNR in dB and the BAD of the pseudo ground-truth (PGT) image and the denoised reconstruction (PSNR and BAD values plotted against each other). For the PSNR measure, we computed the difference (delta) between the values obtained for the reconstructed and the noisy image and here depict averages together with standard errors of the mean (SEM) over three independent executions of the experiment (note that the SEM values are very small and barely visible in the plot and that they were only evaluated for stochastic algorithms). Points are marked in red or blue if the blur effect value of the PGT image is larger or smaller than the blur effect value of the reconstructed image, respectively. Further details are discussed in the text.

As second (more established) benchmark, we considered denoising short exposure TEM images as discussed by Bajic *et al*. [42]. The dataset contains a sequence of noisy short exposure images showing the same scene. As discussed in [42], a low-noise image can be estimated by registering all images to the first image of the sequence and then computing the pixel-wise median values. For our purposes, we adopted this procedure and considered the pixel-wise median as a pseudo ground-truth image. To consider a blind zero-shot setting, we used a single randomly selected image from the dataset for training and testing the algorithms under investigation (see Datasets and S2 Text for details related to the data and the initializations and hyperparameters used, respectively). Due to the availability of a pseudo ground-truth image, we could in this case (and unlike in the case of the SARS-CoV-2 TEM data) evaluate PSNR values. To assess image sharpness, we also utilized the pseudo ground-truth image and calculated its blur absolute difference (BAD) w.r.t. the reconstructed image [56]. To keep information on whether the processed image is sharper or blurrier than the ground-truth image, we have marked points in red or blue to denote whether the blur effect value of the ground-truth image was larger or smaller than the blur effect value of the reconstructed image, respectively. Fig 3 depicts the results of the experiment.

Similarly to the observation made on the SARS-CoV-2 TEM images, S2S achieves the best performance in terms of the noise metric (i.e. PSNR), but the highest value in the blur metric, indicating strongest blur in the reconstructed image. It is the only algorithm that achieves a worse BAD score compared to the noisy image. The second best methods for noise reduction in terms of PSNR are a group of algorithms including DivN, ES3C, BM3D, N2V, N2F, median filtering and PMM, which also show the best BAD scores. In this group, it is noticeable that, firstly, N2F and N2V provide blur effect values close to the respective value of the ground-truth image and, secondly, that DivN and ES3C achieve the highest PSNR values. The Poisson-based methods VST+BM3D and I+VST+BM3D reduce the least noise and achieve the highest BAD scores alongside S2S and the noisy image. Overall, it is evident that the methods assuming additive noise such as S2S, DivN, ES3C, BM3D, N2F and N2V reduce noise more strongly (considering PSNR values) compared to the Poisson-based methods including PMM, VST+BM3D and I+VST+BM3D. Nevertheless, the PSNR and BAD values of the PMM are comparable to the values of the methods assuming additive noise and significantly better than the values of the other Poisson-based methods VST+BM3D and I+VST+BM3D.

### Denoising of fluorescence microscopy images

**Fig 4:**
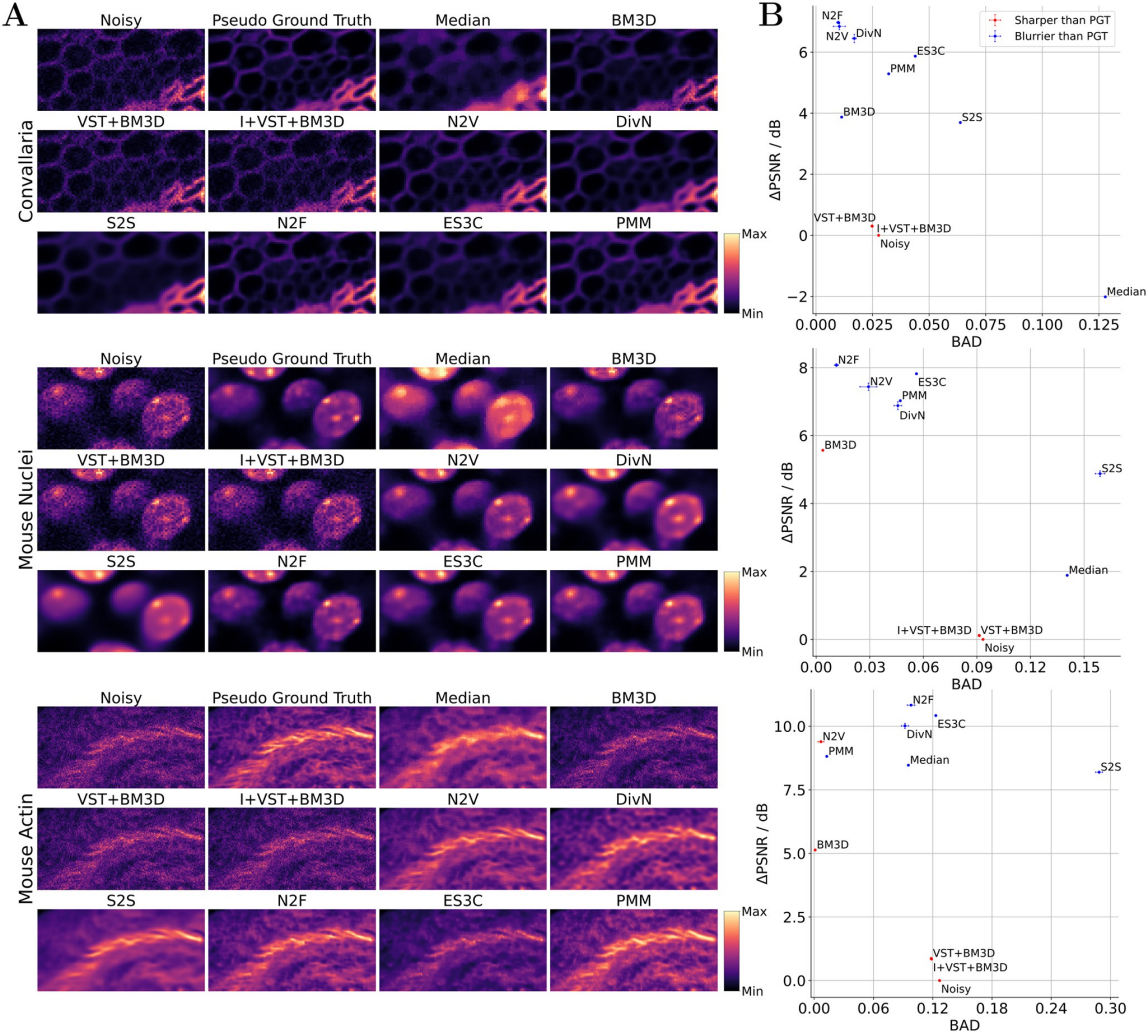
Results for blind zero-shot denoising of fluorescence microscopy images (benchmark adopted from Prakash *et al*. [30]). A: Qualitative comparison of denoised image sections (image sections and colormap chosen to match Fig 2 in [30]; image sections are scaled locally to fill the color range). B: Quantification of the denoising performance in terms of PSNR in dB and the BAD of the pseudo ground-truth (PGT) image and the denoised reconstruction (PSNR and BAD values plotted against each other). For the PSNR measure, we computed the difference (delta) between the values obtained for the reconstructed and the noisy image and here depict averages together with standard errors of the mean (SEM) over three independent executions of the experiment (note that SEM were only evaluated for stochastic algorithms). Points are marked in red or blue if the blur effect value of the PGT image is larger or smaller than the blur effect value of the reconstructed image, respectively. Further details are discussed in the text.

To consider a different microscopy modality, we adopted the recent fluorescence microscopy image denoising benchmark discussed by Prakash *et al*. [30] and applied the algorithms under investigation to the publicly available fluorescence microscopy images FU-PN2V Convallaria, FU-PN2V Mouse Actin, and FU-PN2V Mouse Nuclei. Each of these datasets contains, similarly to the dataset considered in [42], a sequence of different noisy realizations of a static scene for which a denoised reference can be estimated by taking the pixel-wise mean value of all images in the sequence [43]. We adopted the procedure discussed by Prakash *et al*. [30], and used a single randomly selected image from each dataset to train and test all algorithms in a blind zero-shot setting (details under Datasets and S2 Text). The results of the experiment are depicted in Fig 4.

For all three images, the best denoising performance in terms of PSNR values is achieved by N2F, which also achieves the best and second best BAD score for Convallaria and Mouse Nuclei, respectively. The second best methods for noise reduction in terms of PSNR are N2V, DivN, ES3C and PMM, with the ranking of these methods being different for each image. Least noise reduction is achieved by VST+BM3D and I+VST+BM3D. Largest performance differences in terms of PSNR across images can be observed for median filtering: For Mouse Actin, the approach achieves similarly high PSNR values compared to other methods (e.g. PMM) while its performance drops significantly for Mouse Nuclei and Convallaria. According to the BAD metric for all images except Mouse Actin, S2S and median filtering result in the most severe blurring. Similarly to the observations made w.r.t. the PSNR measure, the ranking of ES3C, N2F, N2V, DivN, PMM, BM3D, I+VST+BM3D and VST+BM3D in terms of blur effect measure is not consistent across images. Thereby, BM3D, N2F, N2V and also PMM (for Mouse Actin) achieve the best BAD scores in many cases. In additional control experiments, we observed that the performance of DivN in terms of PSNR could significantly be improved by using external calibration images for noise model estimation and a complete image sequence for training (S4 Table).

## Discussion

We have investigated a variety of recent algorithms that can be applied to blind zero-shot denoising, i.e. to the task of removing noise from a single high resolution microscopy image without requiring additional *a priori* knowledge (such as external training data or noise level information). Among the investigated algorithms, approaches such as median filtering, VST+BM3D, I+VST+BM3D, N2V, N2F, S2S, ES3C and PMM can be applied to a single noisy TEM or FM image relatively directly: Median filtering, and the non-local filtering approaches VST+BM3D and I+VST+BM3D are not data-driven and are applicable without prior training. ES3C and PMM are probabilistic models for unsupervised learning and are by definition trainable on noisy data. N2V, S2S and N2F, on the other hand, employ self-supervised training of DNNs and learn discriminative denoising models based on exclusively noisy training images. The further investigated approaches BM3D and DivN were here, unlike the aforementioned algorithms, executed in conjunction with auxiliary methodology for noise estimation to be applicable to a single noisy image, following procedures used previously (compare Algorithms). The performance of both BM3D and DivN was consequently intertwined with the accuracy of the noise estimation method. That the accuracy of the noise estimation step may turn out to be a crucial factor with potentially decisive impact on the performance for algorithms that rely on such *a priori* information, has repeatedly been discussed (e.g., [57, 58]).

Among the investigated algorithms, those approaches that involve potentially large DNNs with a potentially large number of tunable parameters (e.g. N2V, S2S or DivN), typically increasingly benefit from an increasing amount of training data. N2F plays a particular role in this respect in the sense that it deliberately uses small DNNs which have shown to be advantageous compared to more complex network architectures in blind zero-shot settings in terms of performance and convergence speed [31]. The investigated (non-deep) generative models (i.e. ES3C or PMM) on the other hand typically rely less on the availability of large amounts of training data to learn appropriate representations (similar applies to the considered median or BM3D-based filtering approaches). Here, the data contained in the noisy image itself was observed to be sufficient. The effectiveness of generative models in image restoration tasks has been demonstrated by numerous contributions (e.g. [32, 38, 40, 45, 59, 60]).

For performance evaluations in this study, we focused on two aspects: noise suppression and preservation of image sharpness. To quantify noise suppression, we evaluated signal-to-noise measures, while for image sharpness, we used a no-reference blur metric (cf. Evaluation metrics). The results depicted in Figs 2–4 show that the effectiveness of the investigated algorithms varies across datasets. A potential reason may be given by the match or mismatch between the the assumptions made in the algorithms and the statistical properties of the data, which differ, e.g., depending on the microscopy modality. For example, while methods assuming additive noise including N2V, ES3C, BM3D, and DivN (in the here used variant with GMM-based noise model) performed comparably poorly on the SARS-CoV-2 TEM images in terms of the noise suppression metric, they performed significantly better on the short-exposure TEM dataset as well as on the FM data. In contrast, the Poisson-based methods VST+BM3D and I+VST+BM3D showed to be much more effective on the SARS-CoV-2 data compared to the remaining datasets. These observations may be explained by the different dominant noise components in the individual datasets: While the dominant noise in the SARS-CoV-2 data may be assumed to be well matched by a Poisson distribution, it may, in contrast, be assumed that the dominant noise components in the short-exposure TEM data and the FM data are more closely matched by assuming Gaussian noise (e.g., the digitization noise).

As a further observation, the generative approach PMM performed significantly better on short-exposure TEM and FM data compared to VST+BM3D and I+VST+BM3D, although all three approaches are tailored to Poisson noise. Such an observation may suggest a higher flexibility of the generative approaches in adapting to a noise that may not be exclusively Poisson but a mixture of multiple distributions. In some cases, the performance of PMM was also comparable to N2V, N2F, or DivN, whereby the latter three may be regarded as potentially more suitable approaches for the short exposure TEM and the FM data: N2V, N2F, and DivN assume additive or explicitly Gaussian noise which matches well with the dominant noise source in the short exposure TEM and the FM data.

In control experiments, we observed that algorithm performance could potentially be significantly influenced by varying their hyperparameters. Here, we employed publicly available implementations as far as they were available for the investigated algorithms together with default hyperparameter settings (cf. S2 Text). For approaches such as median filtering, ES3C or PMM, which apply a patch-based processing (cf. S1 Fig), the patch-size showed to be an important hyperparameter (S3 Fig): For instance, we observed that increasingly large patch sizes resulted in many cases with increases in both the SNR and the blur effect measure, which indicates increasingly strong noise suppression but also an increasing loss of image sharpness. Exceptions to this trend could be observed, e.g., on the FM images Convallaria and Mouse Nuclei, for which PSNR values of ES3C, PMM and median filtering partly decreased when increasing the patch size. In general, increasingly large patches imply an increasingly large amount of context information and an increasingly large number of data estimates per image pixel based on which the reconstructed image is computed (cf. S1 Fig). At the same time, the complexity and combinatorics of edges, corners, textures and of other structures that can be captured in a patch grows with the patch size. To adequately capture this increase in complexity and combinatorics of structures in the representations of ES3C or PMM, both the number of patches and the number of model parameters have to be increased. However, the number of extractable patches is defined by the image size and the size of the patches. Increasing the number of patches is only possible to a limited extent in a zero-shot setting, e.g., via data augmentation. Therefore, the representations and the reconstructed images of approaches such as ES3C or PMM become increasingly blurred as the patch size increases. The patch size 6 × 6 employed for the approaches median filtering, ES3C and PMM in Figs 2–4 may consequently be interpreted as a compromise between both of these aspects (amount of context information and complexity and combinatorics of structures in the patches).

In our experiments, increasingly strong noise reduction was often observed in conjunction with an increasing loss of image sharpness and level of detail. Perhaps most illustrative in this respect may be the results on the SARS-CoV-2 TEM images of the S2S approach that here achieved the by far highest noise reduction and the most severe blurring (Fig 2). Evaluating denoising performance solely based on a metric that is indicative about noise suppression may, based on the here reported results, consequently be argued to be suboptimal. Strong noise reduction may turn out to be of little use for image analysis by a virologist if details are lost due to strong blur. In such case, it may be more reasonable to resort to an algorithm that reduces noise less strongly but better preserves image sharpness and level of detail. However, if the task is different, e.g. image segmentation, blurring may no longer be an exclusion criterion, and stronger noise reduction coming at the cost of strong blur could be very beneficial as long as no structures required for segment formation are lost (cf. Fig 1). Algorithm runtime represents a further criterion that is potentially relevant when choosing between blind zero-shot approaches. Here, we observed significant runtime differences when executing the implementations of the investigated methods on the considered datasets (S2 Table). Particularly strong differences could be observed, for instance, when filtering (median and BM3D-based methods) was compared to methods such as S2S or ES3C: On the one hand, we observed for median filtering or BM3D-based methods runtimes on the order of seconds to a few minutes on a conventional CPU, which was comparably fast. On the other hand, S2S was executed for more than a day on a conventional GPU for the short-exposure TEM data, for instance, and ES3C was executed in parallel on multiple high performance CPUs to achieve runtimes below one hour. Among the learning-based algorithms, N2F showed to be by far the fastest method, achieving runtimes almost comparable to those of the BM3D-based approaches.

### Conclusion

The results of this study suggest that the suitability of a blind zero-shot denoising algorithm for given microscopy imaging data is influenced by several factors, including: (i) the match between algorithm assumptions and statistical properties of the data, (ii) the question to which extend an increase in noise suppression at the cost of a loss of image sharpness may be acceptable, and (iii) requirements regarding compute resources and/or time. The algorithms evaluated here showed individual strengths and weaknesses regarding these aspects. The TEM images reveal that S2S has significantly higher noise suppression but also significantly higher blurring compared to the other methods. In the case of FM images, N2F exhibits the most effective denoising performance in terms of PSNR followed by N2V, ES3C and DivN. N2F, N2V (for the TEM dataset cilia and the FM images) and BM3D (for the FM images) were found to be the methods that most closely matched the blur values of the pseudo ground-truth images. Regarding SARS-CoV-2, it is challenging to make a statement without knowledge of the blur effect values of the ground-truth images. N2F and N2V for the Cilia dataset and the FM images, and the Poisson-based methods including VST+BM3D, I+VST+BM3D and PMM for the SARS-CoV-2 data, appear to offer the best trade-off between noise reduction and sharpness preservation. Our study can provide valuable information about practical characteristics of the investigated methods, which may ideally be instructive for choosing the most suitable algorithm for a particular task, e.g., direct analysis by virologists, or downstream image processing tasks such as segmentation. Furthermore, the study provides typical performance characteristics for different approaches that are developed in a number of different subfields, and developments in all the subfields lead to continuous improvements. The diversity of the different methods studied here may also be of interest from another perspective: Especially methods based on different principles and showing strengths in different aspects and for different tasks can potentially be combined very well by meta-approaches such as boosting. One recent example in this direction is a method that combines denoised images from different methods based on a pixel-wise weight map learned from an unsupervised generative network [61], which shows that improved denoising performance can be achieved. Future work may use similar combinations and take denoising as well as sharpness preserving measures into account, or future work may study integrations of, e.g., methods with strong noise reduction and methods with good sharpness preservation. Additionally, it may investigate a combined objective, where optimizing this objective results in strong performance for both criteria.

## Materials and methods

### Datasets

We considered three publicly available microscopy image datasets, two recorded with transmission electron microscopy (TEM) and one with fluorescence microscopy (FM). Dataset-specific details will be provided in the following.

#### Transmission Electron Microscopy Images of SARS-CoV-2 Infected Cell Cultures

Considered were TEM images of ultrathin plastic sections through extracellular SARS-CoV-2 particles in Vero cell cultures [41]. To compile a dataset for our evaluations, we consulted three publicly available repositories [62–64] which provided in total 384 images, from which we selected nine examples (the file names of the selected images are listed in S1 Table). Algorithms were separately applied to each of these images. The TEM images had a resolution of 1032 × 1376 pixels with a pixel size of either 0.64 or 0.54 nm and were stored in 16-bit TIF format; the section thickness was either 65 or 45 nm (see Table 1 of the original publication for details).

#### Short-Exposure TEM Images of Cilia

The second dataset originated from a TEM image denoising benchmark discussed in [42] and consisted of a sequence of 100 noisy short exposure TEM images of a scene showing a cilium. We obtained the original data via personal communication with the authors. The images had a resolution of 2048 × 2048 pixels, and each image depicted a slightly shifted version of the scene. To obtain a less noisy, ground truth-like image, we followed Bajic *et al*. [42] and first aligned all images of the sequence using rigid registration [65] and subsequently computed the pixel-wise median. Finally, we randomly selected one image from the sequence for our evaluations (in our case the 91st image was selected); the randomly selected noisy image and the pseudo ground-truth image are publicly available [66].

#### Fluorescence Microscopy Images

As third example, we considered FU-PN2V Convallaria [43, 67], FU-PN2V Mouse Actin [43] and FU-PN2V Mouse Nuclei [43] fluorescence microscopy image data, which are publicly available [68–70]. In the former two cases, the data consisted of a sequence of 100 images with 1024×1024 pixels, in the latter case of 200 images with 512×512 pixels. To compile a dataset for our evaluations, we followed an earlier suggested procedure and randomly selected one image from each sequence [30] (in our case, the 75th, 41st, and 177th image were selected for Convallaria, Mouse Actin, and Mouse Nuclei, respectively). A ground truth-like image could be obtained by computing the pixel-wise mean of each image sequence [43]. Following publicly available source code associated with this benchmark [71], we employed full-size versions of each test image (which differs to some extend from the methodology applied in [43], in which image sections were considered; personal communication with the authors).

### Algorithms

We investigated in total ten different blind zero-shot denoising approaches, which differed, among other aspects, in their assumptions about the statistical properties of the noise in the data and their noise removal methodology. Assumptions on noise statistics covered, e.g., additivity or signal-dependency of the noise, explicit expressions for the noise distribution (e.g., Gaussian or Poisson), or non-parametric noise models. The applied noise removal methodologies involved local filtering, non-local filtering, discriminative neural networks and probabilistic generative models. Almost all considered algorithms represented established approaches, for which state-of-the-art results on standard denoising benchmarks had been reported in the respective original publications. For our purposes, we executed the algorithms using publicly available implementations (technical details including hyperparameters are described in S2 Text). In the following, we provide a description of the different approaches (also see Table 1 for an overview).

**Table 1:** The noise reduction algorithms considered have different strategies for noise removal and types of noise assumptions. Median filtering is excluded from this table since it has no explicit assumption regarding the noise being additive or signal-dependent. While the DivN approach, in principle, allows for incorporating any suitable imaging noise distribution, here we have follow the publicly available implementation and used a variant of the algorithm in which the noise distribution is described by a GMM. BM3D and ES3C incorporate a Gaussian noise assumption; the VST-based approaches and PMM assume Poisson noise.

	Noise is additive	Noise variance is signal-dependent
Filter-based	BM3D	VST+BM3D I+VST+BM3D
Data-driven	N2V DivN S2S N2F ES3C	PMM

#### Filtering-based Approaches

As most elemementary baseline, we benchmarked median filtering which replaces a given image pixel by the median of the neighboring pixels in a squared window (compare, e.g., [8]). Furthermore, we applied the block-matching and 3D filtering (BM3D [12]) algorithm, and its extensions VST+BM3D [14], and I+VST+BM3D [15]. These algorithms exploit the assumption that natural images exhibit a high degree of image patch self-similarity: For a given patch of a natural image (i.e., a small image section, also referred to as block), further patches at other locations in the same image can be found which contain similar pixel information (typically in, e.g., *l*^2^ sense). After grouping similar patches together, they are processed via filtering. The original BM3D algorithm assumes Gaussian noise and requires *a priori* information on the noise level (i.e., on the standard deviation *σ*). For our purposes, we applied BM3D in conjunction with an auxiliary method for blind noise level estimation, namely the estimator by Chen *et al*. [44] which assumes additive white Gaussian noise. VST+BM3D [14] and I+VST+BM3D [15], in contrast, represent algorithms tailored to Poisson noise. They exploit variance-stabilizing transformations (VST) that enable transforming Poisson noise to a noise that can be treated as Gaussian with unit variance (in which case the classical BM3D can readily be applied). Another filtering approach relevant in the context of blind zero-shot denoising of microscopy images is the Median-Gaussian Filtering Framework for Moiré Pattern Noise Removal [72] in scanning transmission X-ray microscopy images. The approach was not included in our evaluations, however, as to the best knowledge of the authors, no source code has been made available.

#### Discriminative Denoising Neural Networks

As a second type of approaches, we benchmarked Noise2Void (N2V [26]), Self2Self (S2S [29]), and Noise2Fast (N2F [31]), all of which employ self-supervised learning paradigms. N2V employs U-Nets [73], i.e., convolutional neural networks with a U-shaped architecture, which are trained to learn a mapping between pairs of masked noisy image patches. Specifically, the model learns to map a noisy image patch with a subset of the pixels being discarded to the respective unmodified patch. Since both the input and target training data can be obtained from a single noisy image, N2V is applicable in a blind zero-shot setting. The original publication discusses that the blind-spot strategy is likewise applicable for more conventional training settings with clean targets or with paired noisy images; as pointed out in the paper, such training settings provide advantages for the denoising performance compared to training schemes with only a single noisy image [26]. N2V assumes the noise to be additive and conditionally pixel-wise independent given the underlying clean image (the latter of which represents a requirement of the model to avoid learning an identity mapping).

The S2S approach uses DNNs with a bottleneck architecture and partial convolution layers and, similarly to N2V, a form of a blind-spot strategy that enables training on a single noisy image. S2S applies dropout to convolutional layers and image data, which stochastically sets network weights and image pixels to zero, respectively. The masked image and its counterpart, i.e., the image obtained by multiplication with the inverse dropout mask, serve as input and target for the training step. The algorithm computes a restored image by feeding multiple masked versions of a given noisy image through the network and averaging the respective outputs of the model.

N2F uses a convolutional neural network with few hidden layers and as such a comparably simple architecture in relation to N2V and S2S. The training dataset for N2F is derived by downsampling the image to be restored in a way that alternately only preserves even and odd pixels. This process, referred to as ‘chequerboard downsampling’, is applied in both vertical and horizontal direction, resulting in four images which are split into input and target data; the image to be restored is used as validation data. As discussed in the original publication, the training scheme of N2F was primarily optimized for speed. Lequyer *et al*. demonstrate that N2F, at the same time, provides improvements compared to approaches such as N2V or Deep Image Prior (DIP, [59]) also in terms of PSNR and SSIM measures [31].

#### Probabilistic Approaches

We considered two shallow and one deep generative model: Evolutionary Spike-and-Slab Sparse Coding (ES3C [45]), a standard Poisson Mixture Model (PMM), and DivNoising (DivN [30]). ES3C incorporates a sparse coding data model with a spike-and-slab prior [32, 38, 39, 74, 75] that is trained using evolutionary variational strategies. A foundational objective of the approach is to seek compositional features of the data that, when linearly combined, represent the data sufficiently accurately under an additive white Gaussian noise assumption. Sparse Coding (SC [76]), more generally, assumes that data can be accurately recovered using a set of few out of many possible compositional features. SC has been studied intensively for image processing and other tasks (e.g., [40, 77–80]).

Unlike SC, mixture models (such as PMMs) assume a single latent per data point for data generation, i.e., data is modeled in terms of a single compositional feature rather than a combination of multiple features. PMMs assume the observable data to be subject to Poisson distributed noise, and they have been applied in the context of image and audio signal processing applications before (e.g., [81–83]). The here used PMM-based denoising approach is described in more detail in S1 Text.

The DivN approach falls into the category of deep generative models, which can be distinguished from shallow models (such as ES3C or PMM) by their use of DNNs for modeling the data generating process. DivN represents a form of a Variational Autoencoder [84, 85] that was originally studied in the context of image denoising applications. The approach incorporates an model that aims at capturing the noise of the imaging device (i.e., an expression for the distribution of noisy pixels given clean image pixels). The original publication discusses different strategies how such a noise model can be estimated, depending on whether non-noisy or only noisy data is available. One of the former strategies relies on the availability of a sequence of noisy images recorded using the same field of view (i.e., static recording conditions [67]). These images, referred to as calibration data, are used to estimate a non-noisy image by averaging the image sequence. As an alternative for a scenario without calibration data, Prakash *et al*. discuss a strategy referred to as bootstrapping, which leverages auxiliary methodology for estimating a non-noisy image. In both the calibration and the bootstrapping scenario, the noise model is defined in terms of a histogram or a Gaussian mixture model that uses a parameterization depending on both clean and noisy data [43]. Furthermore, Prakash *et al*. discuss a strategy referred to as fully unsupervised which uses a Gaussian noise model with the variance parameterized as a linear function of the modeled clean pixel value [30]. For our purposes, we adapted the examples of the publicly available source code package of DivN and used the bootstrapping method, as the calibration data would violate the blind zero-shot condition. As auxiliary algorithm needed for the bootstrapping method to estimate a non-noisy image, we used N2V. For the learnable noise model of the DivN approach, we chose Gaussian Mixture Models over histograms (also following example code, see S2 Text for further details). Consequently, the variant of DivN that we used in our experiments was tailored to Gaussian noise. Two other related probabilistic approaches include the Stochastically-Connected Random Field algorithm proposed in [7] and the approach developed in [35] which is based on a Poisson-Gaussian Contourlet Hidden Markov model. The former of these algorithms combines random graph and field theory; the latter one is designed for low-count fluorescence microscopy images and models the noise as a combination of Poisson and Gaussian probability distributions. Since, to our best knowledge, no source code has been made publicly available for these algorithm, we have not included them in our evaluations.

### Evaluation metrics

For the TEM images of SARS-CoV-2 infected cell cultures, ‘clean’ reference images were not available, implying that it was not possible to evaluate denoising performance using standard measures such as peak-signal-to-noise ratio (PSNR) or structural similarity (SSIM [86]). Therefore, in this case, we adopted no-reference metrics that could be evaluated based on a single image (compare e.g., [10, 87]). For the short-exposure TEM images of cilia and the FM image data, on the other hand, ‘clean’, i.e. ground truth-like, reference images could be estimated (compare Datasets), which enabled the calculation of PSNR values.

#### Assessment of Noise Suppression

In order to measure noise suppression without ground truth-like reference, we considered the signal-to-noise quantification based on paired hand-labeled signal and background regions as discussed by Bepler *et al*. [28]. Concretely, we followed the procedure described in that publication and labeled *M* pairs of signal and background regions ${\vec{x}}_{s}^{\;(m)}$ and ${\vec{x}}_{b}^{\;(m)}$ for each test image, respectively, to calculate

$$\begin{array}{c} \text{SNR}=\frac{10}{M}\sum_{m=1}^{M}\;{\text{log}}_{10}\left(s^{\left(m\right)}\right)-{\text{log}}_{10}\left({\sigma_{b}^{2}}^{\left(m\right)}\right), \end{array}$$ (1)

with $s^{\left(m\right)}={\left(\mu_{s}^{\left(m\right)}-\mu_{b}^{\left(m\right)}\right)}^{2}$, $\mu_{s}^{\left(m\right)}$ and $\mu_{b}^{\left(m\right)}$ denoting the mean of ${\vec{x}}_{s}^{\;(m)}$ and ${\vec{x}}_{b}^{\;(m)}$, respectively, and ${\sigma_{b}^{2}}^{\left(m\right)}$ denoting the variance of ${\vec{x}}_{b}^{\;(m)}$ (the hand-labeled signal and background regions are depicted in S2 Fig). In the cases with available ground-truth images, PSNRs were computed as follows:

$$\begin{array}{c} \text{PSNR}\left(\text{GT},I\right)=20\;{\text{log}}_{10}\left({\text{MAX}}_{\text{GT}}-{\text{MIN}}_{\text{GT}}\right)-10\;{\text{log}}_{10}\left(\sum_{d=1}^{D}{\left(\text{GT}\left[d\right]-I\left[d\right]\right)}^{2}\right) \end{array}$$ (2)

where MAX_GT_ and MIN_GT_ denote the maximum and minimum pixel amplitudes of the ground-truth image, and GT[d] and I[d] the *d*-th pixel of the ground-truth and the processed image, respectively.

#### Assessment of Image Sharpness

For the quantification of image sharpness, we measured the blur effect as suggested by Crété-Roffet *et al*. [10]. This method analyzes the behavior of neighboring pixels by comparing the original image with a strongly low-pass filtered version of the image (which appears to humans as being significantly blurred). Similarly to the SNR measure by Bepler *et al*., the blur effect measure can be computed based on a single image; the metric quantifies blur perception in terms of a scalar value ranging from zero to one with lower values corresponding to sharper images (i.e., lower blur perception; see the original publication for details). In the cases with available ground-truth images, we computed the blur effect absolute difference (BAD) between the blur effect value of the ground-truth image and the processed image as follows [56]:

$$\begin{array}{c} \text{BAD}=|\text{Blur}\;\text{Effect}(\text{GT})-\text{Blur}\;\text{Effect}(I)| \end{array}$$ (3)

To keep information on whether the processed image is sharper or blurrier than the ground-truth image, we have marked points in red or blue in Figs 3B, 4B and S3B–S3E Fig if the blur effect value of the ground-truth image is larger or smaller than the blur effect value of the processed image, respectively. Evaluations in terms of SSIM values are provided in S3 Table.

## Supporting information

S1 Text Details on Denoising with PMMs. (PDF)

S2 Text Details on Numerical Experiments. (PDF)

S1 File Coordinates of the signal and background regions for the SARS-CoV-2 images. (ZIP)

S2 File Raw measurements of the experiments. This file contains two tables in CSV format. The first table (RawMeasurements_Figs2to4_TabS3_FigS3.csv) presents the blur effect, BAD, SNR, PSNR and SSIM values obtained from experiments conducted across the three datasets using the stated algorithms and settings. The results presented in Figs 2–4, S3 Table and S3 Fig are based on the measurements in this table. The second table (RawMeasurements_TabS4.csv) contains the PSNR values obtained in control experiments with different variants of DivN for the fluorescence microscopy images. The results in S4 Table are based on these PSNR values.
(ZIP)

S1 Fig Illustration of patch-based blind zero-shot image denoising using probabilistic generative models. As input (top left), we use a noisy image (for instance, a TEM image of SARS-CoV-2 infected cell cultures). Using patches extracted from the noisy image (bottom left), we first learn a probabilistic representation of the image using an appropriately chosen data model (e.g. ES3C or PMM). We can then apply the learned representation to probabilistically reconstruct each image patch (bottom right). Finally, we generate a reconstructed image by computing median values of pixel estimates obtained from mutually overlapping patches (top center and right). The image enhancement approach does not require (clean) training data and can directly be applied to a single noisy image (compare Materials and methods in the main text).
(TIFF)

S2 Fig Illustration of the selected TEM images of SARS-CoV-2 infected cell cultures and hand-labeled signal (red) and background (blue) regions. For each TEM image, 20 signal and background regions were labeled, respectively (compare Evaluation metrics in the main text).
(TIFF)

S3 Fig Influence of the patch size hyperparameter on the performance of median filtering, ES3C and PMM. Compared are results for three different patch sizes (3 × 3, 6 × 6 and 12 × 12) for the investigated datasets. Noise suppression and preservation of image sharpness are quantified analogously as in Figs 2–4 in the main text. In subplot A, SNR and blur effect values correspond to averages and standard errors of the mean (SEM) over the nine considered test images. In subplots B–E, PSNR and BAD values of PMM and ES3C correspond to averages and SEM values of three independent runs of the algorithm (note that the SEM values are so small that they are hardly visible). The trend of the algorithms across the three different patch sizes is represented by the dashed lines.
(TIFF)

S1 Table File names of the selected TEM images of SARS-CoV-2 infected cell cultures. (TIFF)

S2 Table Hardware and approximate runtime used to execute the implementations of the investigated algorithms on the considered datasets. The runtimes were not measured with the aim of enabling a systematic comparison, but to indicate an approximate order of magnitude.
(TIFF)

S3 Table Structural similarity index (SSIM) values for the denoised TEM images of Cilia and the FM images of Convallaria, Mouse Nuclei and Mouse Actin of Figs 3 and 4 in the main text. For the stochastic approaches (algorithms listed below, including, N2V in the table), we performed three independent executions of each experiment and here list averages over these runs; standard deviations were smaller or equal 0.01. The bold numbers denote the best SSIM value for each dataset.
(TIFF)

S4 TablePSNR values (in dB) obtained with different variants of DivN for the considered fluorescence microscopy images.For the results of Fig 4 in the main text, we executed DivN using N2V-based bootstrapping for noise model estimation and a single noisy image for training (compare Materials and methods in the main text). In further control experiments, we also investigated applications of DivN with calibration data for noise model estimation and training on full image series. For calibration, we used publicly available calibration images [68–70]. In total, we performed four different types of experiments per image, which we here refer to as ${\text{DivN}}_{\text{C}}^{1}$, ${\text{DivN}}_{\text{B}}^{1}$, ${\text{DivN}}_{\text{C}}^{\text{all}}$, and ${\text{DivN}}_{\text{B}}^{\text{all}}$. The superscripts ^1^ and ^all^ denote the variants of the algorithm that use training on a single and on all images of a given dataset, respectively; the subscripts _C_ and _B_ indicate noise model estimation using calibration data and bootstrapping, respectively. The table lists averages and standard deviations over three executions of the algorithm per setting.(TIFF)
