# Supplementary material for: Zero-shot denoising of microscopy images recorded at high-resolution limits
Source: PLoS Comput Biol. 2024 Jun 10;20(6):e1012192. doi: 10.1371/journal.pcbi.1012192 (PMC11230634; doi:10.1371/journal.pcbi.1012192)
Supplement: S1 Text — (PDF) [file pcbi.1012192.s001.pdf]

S1 Details on Denoising with PMMs

In order to enhance a microscopy image with PMMs, we extract overlapping patches [1–4] from the image and treat the obtained set of image patches as our training dataset $\mathcal{Y} = \{\vec{y}^{\,(n)}\}_{n=1}^{N}$ (patch sizes used are listed in S2 Text). Given this training dataset, we seek parameters $\Theta^* = \mathrm{argmax}_\Theta\, \mathcal{L}(\Theta)$ that optimize the data log-likelihood. A direct optimization is usually challenging, and, instead, efficient algorithms use, for instance, Expectation Maximization approaches [5, 6] and optimize a lower bound (also known as free energy or evidence lower bound – ELBO)

$$\mathcal{F}(q,\Theta) \leq \mathcal{L}(\Theta) = \sum_{n=1}^{N} \log p(\vec{y}^{\,(n)} \mid \Theta) = \sum_{n=1}^{N} \log \sum_{c=1}^{C} p(\vec{y} \mid c, \Theta) p\,(c \mid \Theta) \tag{S1.1}$$

of the log-likelihood. $p\,(c \mid \Theta)$ and $p\,(\vec{y} \mid c, \Theta)$ are referred as prior and noise model, respectively, given by

$$p\,(c \mid \Theta) = \pi_c, \qquad \text{and} \qquad p\,(\vec{y} \mid c, \Theta) = \prod_{d=1}^{D} \mathrm{Poiss}\,(y_d; W_{cd})\,, \tag{S1.2}$$

where $\vec{\pi} = \{\pi_c\}_{c=1}^{C}$ denote the components' prior activations, for which $\pi_c \in [0, 1]$ and $\sum_{c=1}^{C} \pi_c = 1$ applies; $W = \{\vec{W}_c\}_{c=1}^{C}$ denote cluster centers with $\vec{W}_c = \{W_{cd}\}_{d=1}^{D}$ and C denotes the number of cluster components. $\mathcal{F}(q,\Theta)$ is iteratively and alternatingly optimized w.r.t. the variational distributions $q = \{q^{(n)}\}_{n=1}^{N}$ (E-step) and the model parameters Θ (M-step). In order to turn the inequality in Eq (S1.1) into an equality such that the lower bound matches the likelihood, the functionals $q^{(n)}$ are chosen to match the exact posterior in the E-step:

$$p(c \mid \vec{y}, \Theta) = \frac{p(\vec{y} \mid c, \Theta) p\,(c \mid \Theta)}{\sum_{c'=1}^{C} p(\vec{y} \mid c', \Theta) p(c' \mid \Theta)}. \tag{S1.3}$$

In the M-step, the parameters W_{cd} and π_c are updated using the following update equations

$$W_{cd} = \frac{\sum_{n=1}^{N} p(c|\vec{y}^{\,(n)}, \Theta) y_d^{(n)}}{\sum_{n=1}^{N} p(c|\vec{y}^{\,(n)}, \Theta)}, \qquad \pi_c = \frac{1}{N} \sum_{n=1}^{N} p(c \mid \vec{y}^{\,(n)}, \Theta). \tag{S1.4}$$

Given a set of optimized parameters Θ, we can use the learned data representation under the PMM model for probabilistic data estimation: for instance, we can estimate the most likely, non-noisy version $\vec{y}^{\,\mathrm{est}}$ of a given noisy data point $\vec{y}$ by estimating the first moment of the modeled pixel distribution. Here, we adapt the line of reasoning used in [7], and derive a data estimator based on the posterior predictive distribution $p(\vec{y}^{\,\mathrm{est}}|\vec{y}, \Theta)$. For the PMM of Eq (S1.2), the posterior predictive distribution can be written as follows:

$$p(\vec{y}^{\,\mathrm{est}}|\vec{y}) = \sum_{c} p(\vec{y}^{\,\mathrm{est}}|c, \Theta) p(c|\vec{y}, \Theta). \tag{S1.5}$$

The noisy pixel values are then replaced by the expectation values of this posterior predictive distribution:

$$y_d^{\mathrm{est}} \leftarrow \mathbb{E}_{p(\vec{y}^{\,\mathrm{est}}|\vec{y},\Theta)}\left[y_d^{\mathrm{est}}\right] = \mathbb{E}_{p(c|\vec{y},\Theta)}\left[\mathbb{E}_{p(\vec{y}^{\,\mathrm{est}}|c,\Theta)}\left[y_d^{\mathrm{est}}\right]\right], \tag{S1.6}$$

where $\mathbb{E}$ denotes expectation and $\mathbb{E}_{p(x)}\left[g(x)\right] = \sum_x p(x) g(x)$ for discrete x with $\sum_x$ running over all possible configurations of x. The inner expectation is the first moment

of the noise model (Eq (S1.2), right). This expectation is given by $\mathbb{E}_{p(\vec{y}^{\,\mathrm{est}}|c,\Theta)}\big[y_d^{\mathrm{est}}\big] = W_{cd}$. Inserting this into Eq (S1.6), the data estimator yields to:

$$\mathbb{E}_{p(\vec{y}^{\,\mathrm{est}}|\vec{y},\Theta)}\big[y_d^{\mathrm{est}}\big] = \mathbb{E}_{p(c|\vec{y},\Theta)}\big[W_{cd}\big]. \tag{S1.7}$$

We apply Eq (S1.7) to each data point in $\mathcal{Y}$ (i.e., to each image patch). Due to mutually overlapping patches, this results in multiple denoised estimates for a given image pixel, and, here, we consider median values of pixel estimations for image reconstruction (see S1 Fig for an illustration of the pipeline). Note that this pipeline is not specific to PMM but can be adopted to other generative models (compare [7]; the here reported results of ES3C were obtained with the same procedure).

References

1. Elad M, Aharon M. Image Denoising Via Sparse and Redundant Representations Over Learned Dictionaries. IEEE Transactions on Image Processing. 2006;15(12):3736–3745.

2. Mairal J, Elad M, Sapiro G. Sparse Representation for Color Image Restoration. IEEE Transactions on Image Processing. 2008;17(1):53–69.

3. Zhou M, Chen H, Paisley J, Ren L, Li L, Xing Z, et al. Nonparametric Bayesian Dictionary Learning for Analysis of Noisy and Incomplete Images. IEEE Transactions on Image Processing. 2012;21(1):130–144.

4. Burger HC, Schuler CJ, Harmeling S. Image denoising: Can plain Neural Networks compete with BM3D? In: IEEE Conference on Computer Vision and Pattern Recognition; 2012. p. 2392–2399.

5. Saul LK, Jordan MI. Exploiting Tractable Substructures in Intractable Networks. In: Advances in Neural Information Processing Systems. vol. 8; 1995.

6. Neal R, Hinton G. A View of the EM Algorithm that Justifies Incremental, Sparse, and other Variants. Learning in Graphical Models. 1998; p. 355–368.

7. Drefs J, Guiraud E, Lücke J. Evolutionary Variational Optimization of Generative Models. Journal of Machine Learning Research. 2022;23(21):1–51.
