# Supplementary material for: Zero-shot denoising of microscopy images recorded at high-resolution limits
Source: PLoS Comput Biol. 2024 Jun 10;20(6):e1012192. doi: 10.1371/journal.pcbi.1012192 (PMC11230634; doi:10.1371/journal.pcbi.1012192)
Supplement: S2 Text — (PDF) [file pcbi.1012192.s002.pdf]

S2 Details on Numerical Experiments

Data

S1 Table lists the file names of the nine TEM Images of SARS-CoV-2 infected cell cultures that we selected for our evaluations. S2 Fig depicts the signal and background regions used for the signal-to-noise quantification in Fig 2B.

Implementations and hyperparameters

The following list gives details about the source codes used for the algorithms we compare. All used software sources are publicly available except of PMM, which is an implementation of a standard Poisson mixture model made available together with this paper. Following the respective instructions, it was straightforward to obtain, install, and execute the algorithms using relatively standard software setups (Linux distributions, python, MATLAB, etc.).

Median Filtering. We used the implementation of a median filter provided by the SciPy (ndimage) package [1].

BM3D. We used the official [2] BM3D Python software and executed the algorithm using the configuration 'stage_arg=BM3DStages.ALL_STAGES'. To provide a noise level estimate to the algorithm, we used the method of Chen et al. [3] and the respective publicly available implementation [4] together with default hyperparameters.

VST+BM3D. We used the official [5] VST+BM3D MATLAB software and executed the algorithm with the configuration of the 'demo_Poisson_experiments_table' example available in the file package.

I+VST+BM3D. We used the official [6] I+VST+BM3D MATLAB software and executed the algorithm with the configuration of the 'demo_iterVSTpoisson' example available in the file package.

N2V. We used the official [7] Noise2Void implementation and executed the algorithm with the configuration of the 'BSD68_reproducibility' example available in the respective GitHub repository.

DivN. We used the official [8] DivNoising implementation and executed the algorithm with the configuration of the 'Convallaria' and 'Mouse_nuclei' example available in the respective GitHub repository. For the Mouse Actin, Cilia and SARS-CoV-2 experiments, we adopted the settings of the Convallaria example. To create a noise model, we used the bootstrapping method based on the respective denoised outcome of the N2V algorithm (following the example code).

S2S. We used the official [9] Self2Self implementation and executed the algorithm with the configuration of the 'demo_denoising' script available in the respective GitHub repository. We set the dropout rate to 0.3 (as suggested in examples in the 'demo_denoising' script), and the parameters 'sigma' and 'is_realnoisy' to '-1' and 'True', respectively. We modified the preprocessing pipeline by dividing the pixel amplitudes with the maximum pixel amplitude of a given input image rather than with the value 255 (compare 'utils.py/ line 26'). Accordingly, we modified the postprocessing pipeline by multiplying the pixel amplitudes of the output image with the previously determined

maximum value and then saved the image with single precision (compare 'demo_denoising.py/ line 56').

N2F. We used the official [10] Noise2Fast implementation and executed the algorithm with the configuration of the 'N2F' script available in the respective GitHub repository.

ES3C. We used the official [11] ES3C implementation and adapted the 'image-denoising' example available in the respective GitHub repository for our purposes. Hyperparameters were set as follows: 'patch_height=6', 'H=512', 'Ksize=30', 'parent_selection=fit', 'mutation_algorithm=randflip', 'no_parents=20', 'no_children=1', 'no_generations=1', 'no_epochs=20'. We executed the implementation in parallel on 60 Intel Xeon Platinum 9242 CPUs.

PMM. We used our own implementation [12] of a standard Poisson mixture model trained with Expectation Maximization, and data estimation analogous to ES3C for denoising (see S1 Text for details). To train PMM on the microscopy images, we used a patch size and a cluster size of $D = 6 \times 6$ (same value as used for ES3C) and $C = 1000$, respectively. PMM was trained for 100 epochs for each image. We initialized PMM as follows: Priors $\{\pi_c^{\mathrm{init}}\}_{c=1}^{C}$ were uniformly randomly drawn from the interval $(0, 1)$. The columns of the matrix W^{init} were initialized with the centers found by running the kmeans++ algorithm on the patch data. We executed the implementation in parallel on an Intel Xeon 4214 CPU.

Runtimes

S2 Table lists the hardware we used to execute the implementations of each algorithm and provides an overview of their approximate runtimes in our experiments.

References

1. Virtanen P, Gommers R, Oliphant TE, Haberland M, Reddy T, Cournapeau D, et al. SciPy 1.0: Fundamental Algorithms for Scientific Computing in Python. Nature Methods. 2020;17:261–272.

2. Mäkinen Y. Python wrapper for BM3D denoising. *Python Package Index*; Accessed 2020-04-18. `https://pypi.org/project/bm3d`.

3. Chen G, Zhu F, Ann Heng P. An Efficient Statistical Method for Image Noise Level Estimation. In: IEEE International Conference on Computer Vision; 2015. p. 477–485.

4. Chen G, Zhu F, Ann Heng P. Noise Level Estimation for Signal Image. *GitHub repository*; Last accessed: 2021-12-01. `https://github.com/zsyOAOA/noise_est_ICCV2015`.

5. Mäkitalo M, Foi A. Denoising software for Poisson and Poisson-Gaussian data; Last accessed: 2023-04-19. `https://webpages.tuni.fi/foi/invansc/`.

6. Azzari L, Foi A. Iterative Poisson image denoising software; Last accessed: 2023-04-19. `https://webpages.tuni.fi/foi/invansc/`.

7. Krull A, Buchholz TO, Jug F. Noise2Void - Learning Denoising from Single Noisy Images. *GitHub repository*; Last accessed: 2020-06-10. `https://github.com/juglab/n2v`.

8. Prakash M, Krull A, Jug F. DivNoising: Diversity Denoising with Fully Convolutional Variational Autoencoders. *GitHub repository*; Last accessed: 2021-10-20. https://github.com/juglab/DivNoising.

9. Quan Y, Chen M, Pang T, Ji H. Self2Self With Dropout: Learning Self-Supervised Denoising From Single Image. *GitHub repository*; Last accessed: 2021-10-19. https://github.com/scut-mingqinchen/self2self.

10. Lequyer J, Philip R, Sharma A, Hsu WH, Pelletier L. A fast blind zero-shot denoiser. *GitHub repository*; Last accessed: 2023-12-08. https://github.com/pelletierlab/Noise2Fast.

11. EVO developers. EVO - Evolutionary Variational Optimization of Generative Models. *GitHub repository*; Last accessed: 2022-06-22. https://github.com/tvlearn/evo.

12. Salwig S. Denoising with Poisson Mixture Models. *GitHub repository*; Last accessed: 2024-04-29. https://github.com/salwig/pmm.
