# Supplementary figures and images for: Zero-shot denoising of microscopy images recorded at high-resolution limits
Source: PLoS Comput Biol. 2024 Jun 10;20(6):e1012192. doi: 10.1371/journal.pcbi.1012192 (PMC11230634; doi:10.1371/journal.pcbi.1012192)

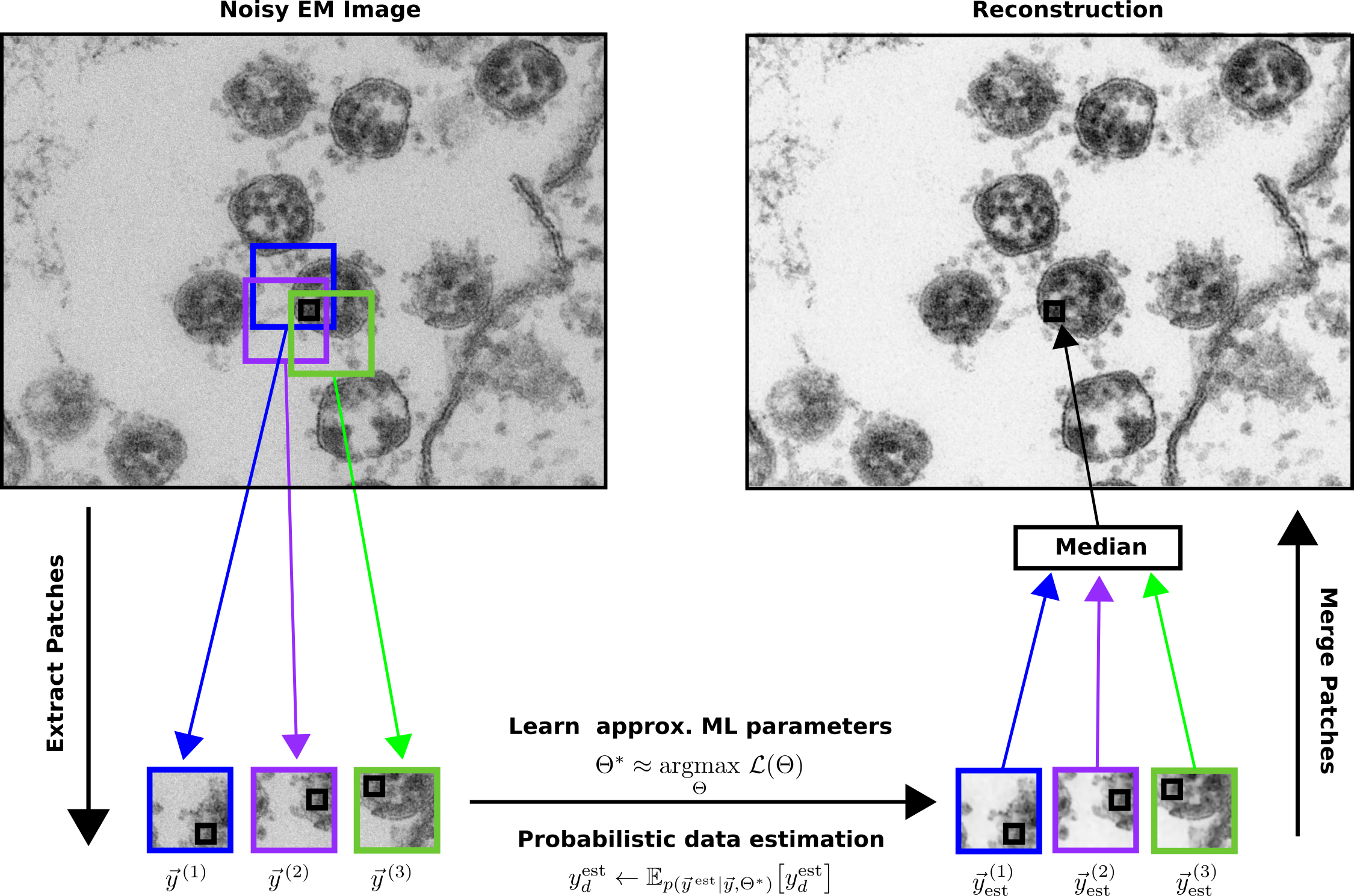

Supplement: S1 Fig — As input (top left), we use a noisy image (for instance, a TEM image of SARS-CoV-2 infected cell cultures). Using patches extracted from the noisy image (bottom left), we first learn a probabilistic representation of the image using an appropriately chosen data model (e.g. ES3C or PMM). We can then apply the learned representation to probabilistically reconstruct each image patch (bottom right). Finally, we generate a reconstructed image by computing median values of pixel estimates obtained from mutually overlapping patches (top center and right). The image enhancement approach does not require (clean) training data and can directly be applied to a single noisy image (compare Materials and methods in the main text). (TIFF) [file pcbi.1012192.s005.tiff]

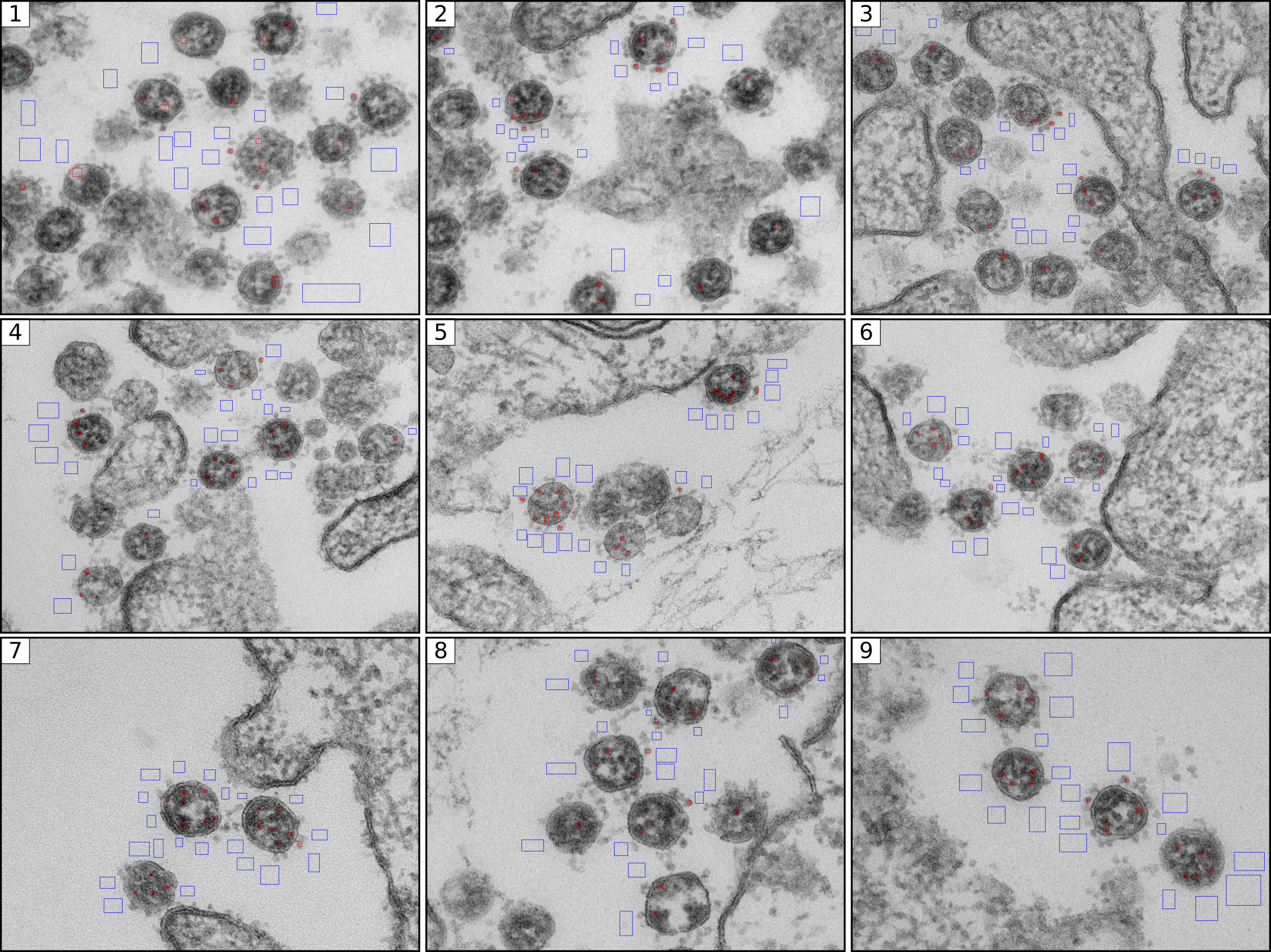

Supplement: S2 Fig — For each TEM image, 20 signal and background regions were labeled, respectively (compare Evaluation metrics in the main text). (TIFF) [file pcbi.1012192.s006.tiff]

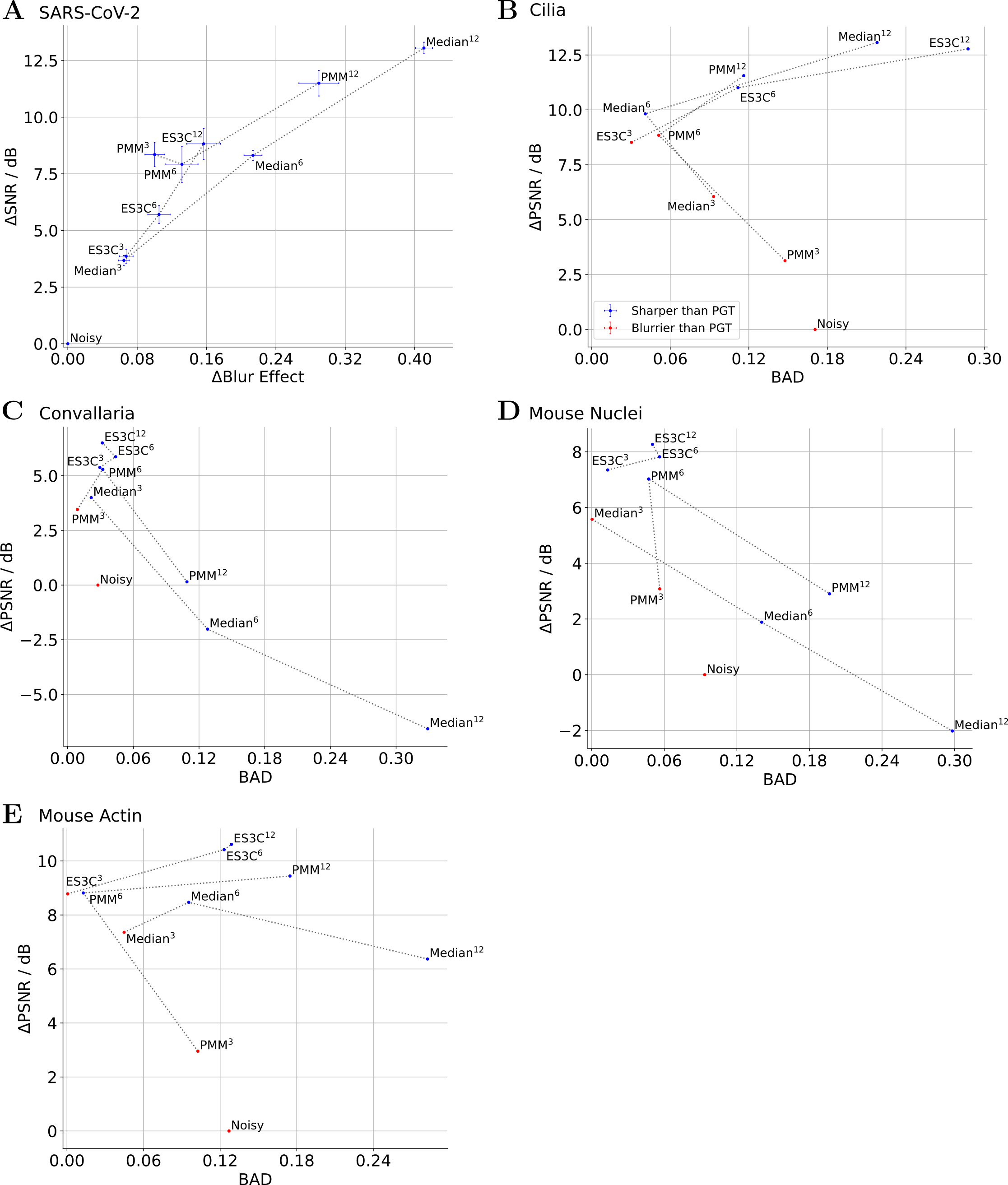

Supplement: S3 Fig — Compared are results for three different patch sizes (3 × 3, 6 × 6 and 12 × 12) for the investigated datasets. Noise suppression and preservation of image sharpness are quantified analogously as in Figs 2–4 in the main text. In subplot A, SNR and blur effect values correspond to averages and standard errors of the mean (SEM) over the nine considered test images. In subplots B–E, PSNR and BAD values of PMM and ES3C correspond to averages and SEM values of three independent runs of the algorithm (note that the SEM values are so small that they are hardly visible). The trend of the algorithms across the three different patch sizes is represented by the dashed lines. (TIFF) [file pcbi.1012192.s007.tiff]

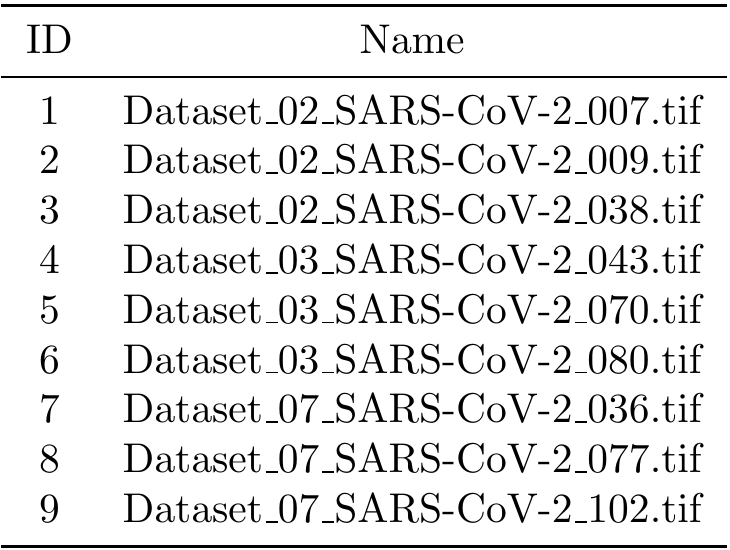

Supplement: S1 Table — (TIFF) [file pcbi.1012192.s008.tiff]

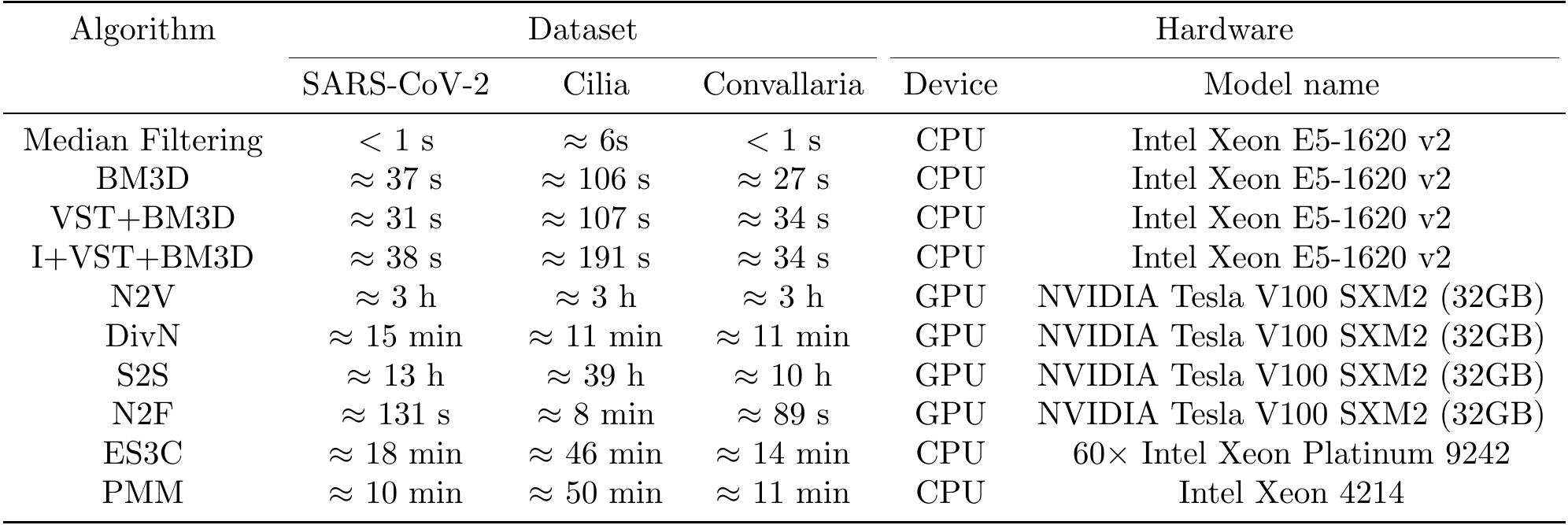

Supplement: S2 Table — The runtimes were not measured with the aim of enabling a systematic comparison, but to indicate an approximate order of magnitude. (TIFF) [file pcbi.1012192.s009.tiff]

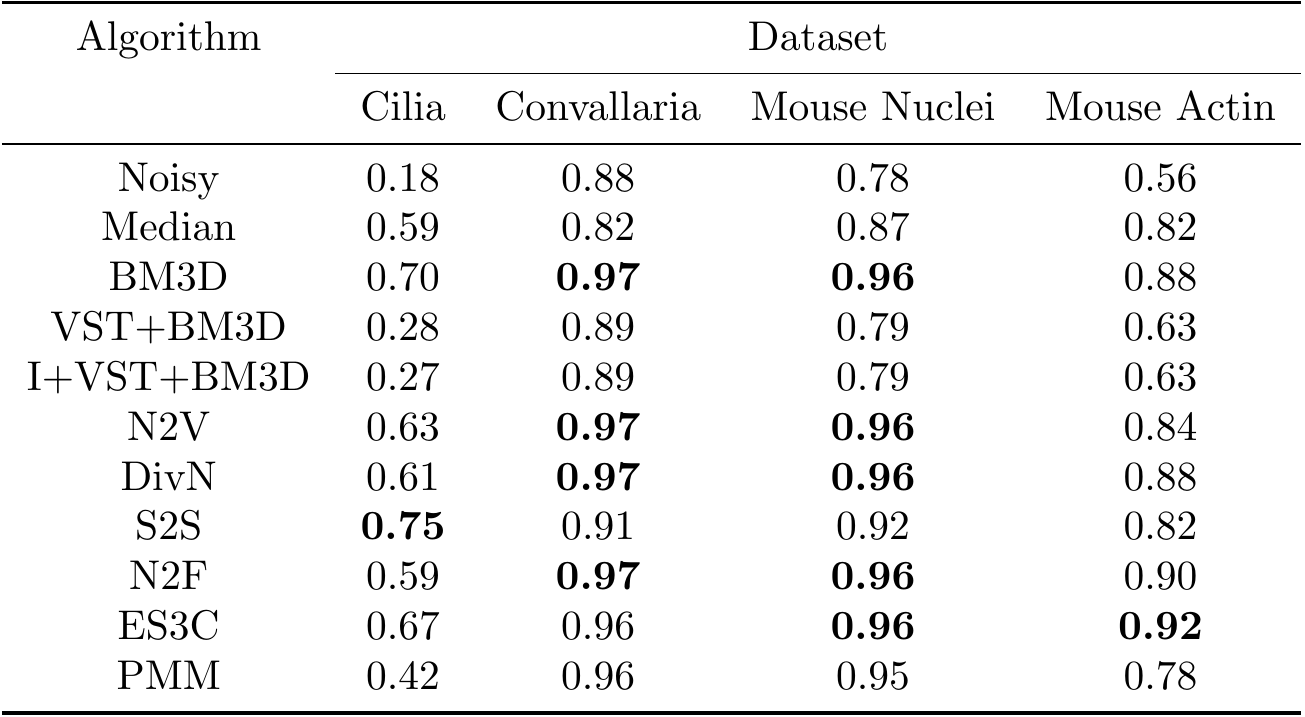

Supplement: S3 Table — For the stochastic approaches (algorithms listed below, including, N2V in the table), we performed three independent executions of each experiment and here list averages over these runs; standard deviations were smaller or equal 0.01. The bold numbers denote the best SSIM value for each dataset. (TIFF) [file pcbi.1012192.s010.tiff]

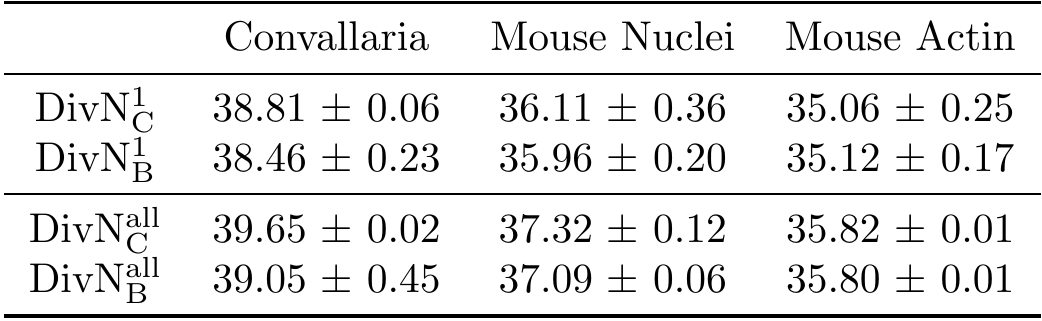

Supplement: S4 Table — For the results of Fig 4 in the main text, we executed DivN using N2V-based bootstrapping for noise model estimation and a single noisy image for training (compare Materials and methods in the main text). In further control experiments, we also investigated applications of DivN with calibration data for noise model estimation and training on full image series. For calibration, we used publicly available calibration images [68–70]. In total, we performed four different types of experiments per image, which we here refer to as DivNC1, DivNB1, DivNCall, and DivNBall. The superscripts 1 and all denote the variants of the algorithm that use training on a single and on all images of a given dataset, respectively; the subscripts C and B indicate noise model estimation using calibration data and bootstrapping, respectively. The table lists averages and standard deviations over three executions of the algorithm per setting. (TIFF) [file pcbi.1012192.s011.tiff]
